# Osteosarcoma cell intrinsic PD-L2 signals promote invasion and metastasis via the RhoA-ROCK-LIMK2 and autophagy pathways

**DOI:** 10.1038/s41419-019-1497-1

**Published:** 2019-03-18

**Authors:** Tingting Ren, Bingxin Zheng, Yi Huang, Shidong Wang, Xing Bao, Kuisheng Liu, Wei Guo

**Affiliations:** 1 Musculoskeletal Tumor Center, Peking University People’s Hospital, Beijing, People’s Republic of China; 2 Beijing Key Laboratory of Musculoskeletal Tumor, Beijing, People’s Republic of China; 3 Department of Orthopedic Surgery, The Affiliated Hospital of Qingdao University, Qingdao, People’s Republic of China

## Abstract

Known as co-stimulatory molecule, programmed death ligand-2 (PD-L2) contributes to T-cell exhaustion by interaction with programmed death-1 (PD-1) receptor, but its tumor cell-intrinsic signal effects have been little investigated. PD-L2 expression was detected by immunohistochemistry in 18 pairs of primary osteosarcoma tissues and matching lung metastasis tissues. We also investigated the effects of PD-L2 knockdown on osteosarcoma both in vitro and in vivo. In our study, PD-L2 expression was elevated in lung metastases compared with primary osteosarcoma according to an immunohistochemistry assay. Wound-healing and transwell assays revealed that PD-L2 knockdown  leaded to inhibition of migration and invasion of human osteosarcoma cells in vitro. Mechanistically, we demonstrated that PD-L2 knockdown attenuated migration and invasion by inactivating RhoA-ROCK-LIMK2 signaling, suppressing epithelial–mesenchymal transition (EMT), and inhibiting autophagy by decreasing beclin-1 expression. In support of these observations, beclin-1 knockdown also inhibited activation of the RhoA-ROCK-LIMK2 pathway, leading to autophagy inhibition-induced blockade of migration and invasion. Depletion of PD-L2 in KHOS cells markedly weakens pulmonary metastatic potential in vivo by orthotopic transplantation of nude mice. Our study reveals a pro-metastatic functional mechanism for PD-L2 in osteosarcoma. Furthermore, we demonstrate a regulatory role for PD-L2 on autophagy, as well as a relationship between autophagy and metastasis in osteosarcoma, which may represent a potential therapeutic target for osteosarcoma.

## Introduction

Osteosarcoma is the most common primary malignant bone tumor in teenagers^1,2^, exhibiting early metastasis with poor prognosis^3^. There have been no significant improvements in treatment for osteosarcoma in recent decades and the current mainstream treatment remains neoadjuvant chemotherapy combined with surgery. However, discovery of novel chemotherapeutic agents for osteosarcoma has plateaued and there are currently no target-specific drugs available for osteosarcoma. Thus, a new treatment with increased efficacy is urgently needed, particularly for metastatic osteosarcoma.

In recent years, immune checkpoint inhibitor (ICI), as represented by the programmed cell death-1 (PD-1) monoclonal antibody, has been shown to have efficacious therapeutic benefit in many solid tumors by restoring the immune function of T-cells to kill tumor cells. The ligands of the PD-1 receptor include programmed death ligand-1 (PD-L1) and PD-L2, and their interaction attenuates T-cell antitumor effects, resulting in immune escape^4,5^.

Due to ICI’s promising therapeutic effects, most studies have focused on communication between tumor cells and T-cells. However, few studies have been conducted on the tumor cell-intrinsic signaling of PD-L1 and PD-L2. Recent findings^6,7^ have reported that a minor subset of patients treated with PD-L1/PD-1 mAb therapy responded with rapid disease progression patterns. One reason for this may be the PD-1/PD-L1 axis-mediated inherent functions in tumor cells and PD-1/PD-L1 blockade may affect the tumor cell-intrinsic signaling network, enhancing tumor growth or progress. This suggests that ICI treatment effects may be associated with tumor cell-intrinsic signaling of PD-L1 and PD-L2. Previous studies have demonstrated that PD-L1 and PD-L2 are correlated with multiple tumor phenotypes, including epithelial–mesenchymal transition (EMT), proliferation, and autophagy^8–11^. The current study indicates that PD-L1 mRNA expression is detected in osteosarcoma^12^. Metastatic, but not primary, osteosarcoma tumors express PD-L1^13,14^, whereas recent studies show that PD-L1 is detected in primary osteosarcoma, with no significant differences between primary and metastatic osteosarcoma^15,16^. Moreover, PD-L1 may be correlated with immune suppression, cisplatin resistance, and metastasis-related pathway activation in osteosarcoma by datamining and bioinformatics analyses^16^. Compared with PD-L1, the functional significance of PD-L2 in tumor cells has been scarcely investigated. To our knowledge, there is no relevant literature reporting on the tumor intrinsic signaling effects of PD-L2 in osteosarcoma.

In this study, PD-L2 expression was measured in primary and metastatic osteosarcoma. The roles of PD-L2 in osteosarcoma cell migration, invasion, and autophagy were investigated both in vitro and in vivo. In addition, we explored the underlying mechanisms of tumor expansion and metastasis mediated by PD-L2.

## Results

### PD-L2 expression is elevated in lung metastases of osteosarcoma

**Fig. 1:**
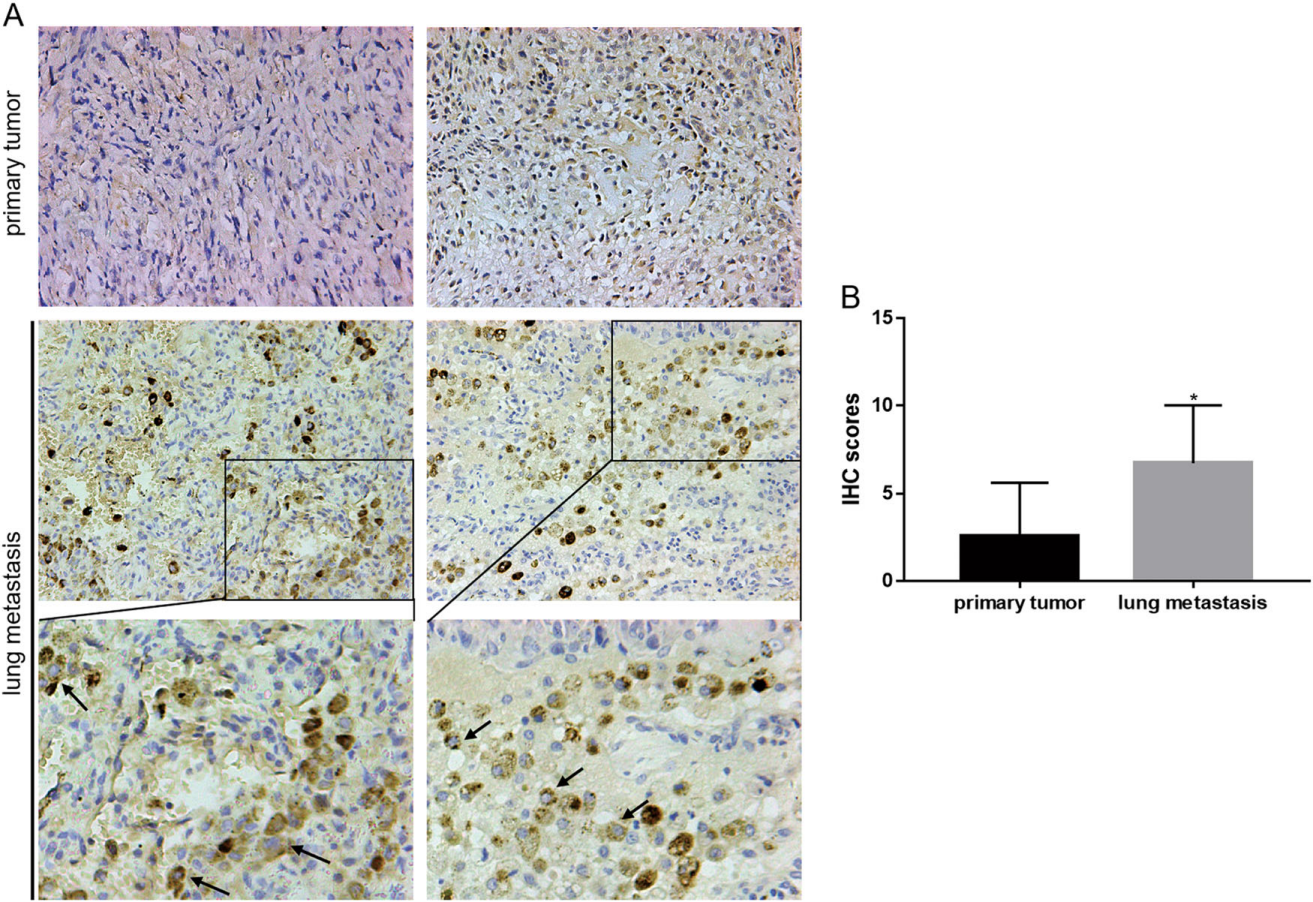
Elevated PD-L2 expression in osteosarcoma lung metastasis. **a** PD-L2 expression in 18 pairs of primary osteosarcoma tissues and matching lung metastasis tissues was detected by IHC. Representative images are shown (magnification at × 200 and × 400). Arrows indicate membrane and cytoplasmic expressions. **b** IHC total score of PD-L2 staining were analyzed between primary osteosarcoma and matching lung metastasis groups. Data are presented as the mean ± SD. *P < 0.05

Immunohistochemistry (IHC) analysis of PD-L2 was performed on 18 pairs of primary osteosarcoma samples and matching lung metastasis samples. PD-L2 exhibited membranous and cytoplasmic expression (Fig. 1a), and we observed that PD-L2 expression was increased in lung metastasis tissues compared with primary osteosarcoma tissues (Fig. 1b), suggesting that PD-L2 may have a crucial role in osteosarcoma metastasis.

### PD-L2 knockdown inhibits migration and invasion of osteosarcoma cells

**Fig. 2:**
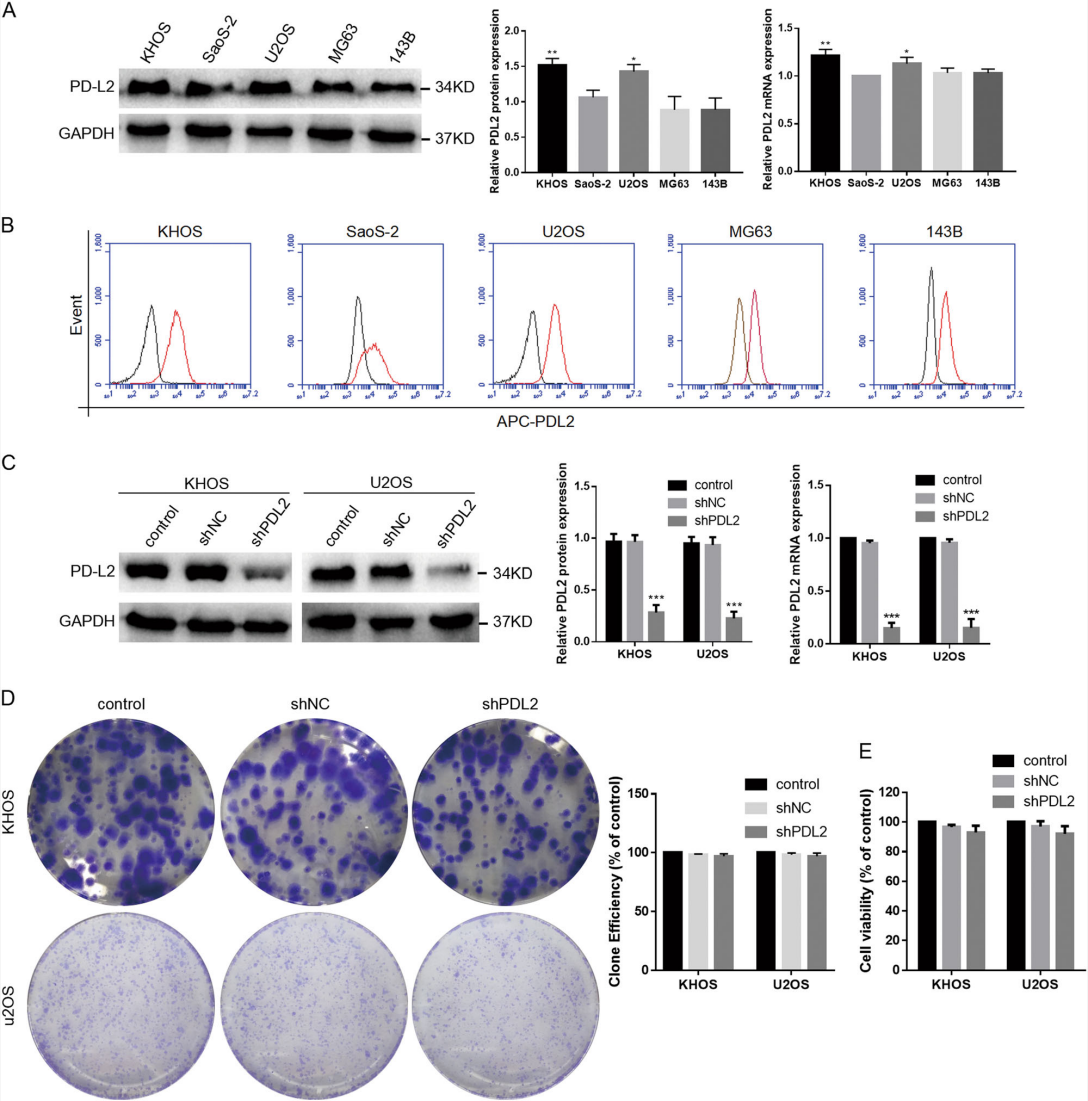
Effect of PD-L2 expression on the proliferation of osteosarcoma cells. **a** PD-L2 expression in osteosarcoma cell lines was evaluated by western blotting and real-time PCR. **b** The osteosarcoma cell lines (red) exhibited differing degrees of PD-L2 expression compared with the isotype control (black) by flow cytometry. **c** KHOS and U2OS cells were stably transfected with shPD-L2 or shNC lentivirus, following evaluation of PD-L2 expression by western blotting and real-time PCR. **d** Cell proliferation of KHOS and U2OS cells in response to PD-L2 knockdown determined by cell colony formation assay. **e** Cell viability of KHOS and U2OS cells after PD-L2 knockdown assayed by CCK-8. All experiments were repeated three times. Data are presented as the mean ± SD. *P < 0.05, **P < 0.01, ***P < 0.001

The PD-L2 mRNA and protein levels were examined in osteosarcoma cell lines, among which KHOS and U2OS exhibited significantly higher levels of PD-L2 mRNA and protein than SaoS-2 cells (Fig. 2a). Flow cytometric analyses were also performed on osteosarcoma cell lines for protein detection, the results revealed that these osteosarcoma cell lines exhibited differing degrees of PD-L2 expression compared with the isotype control (Fig. 2b). As such, the KHOS and U2OS cell lines were chosen for further study. To examine the effect of PD-L2 on proliferation, migration, and invasion of osteosarcoma cells, KHOS and U2OS cells were transfected with short hairpin RNA (shRNA) targeting PD-L2, and PD-L2 expression was subsequently detected by western blotting (WB) and real-time PCR (Fig. 2c). In addition, the expression of PD-L1 remained with no changes after PD-L2 knockdown in KHOS and U2OS cells, which was detected by WB (Additional File 1A).

**Fig. 3:**
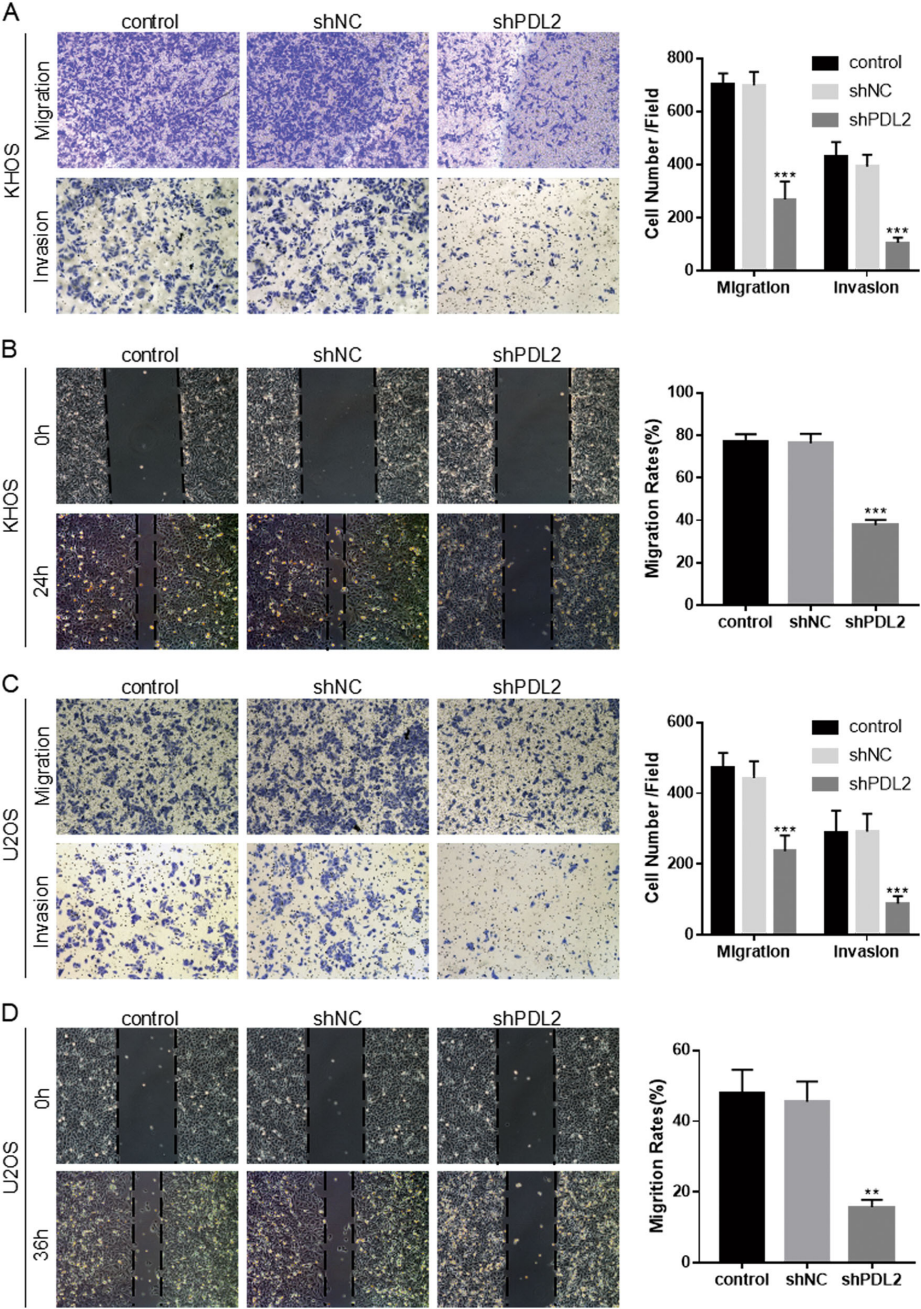
PD-L2 knockdown inhibits migration and invasion of osteosarcoma cells. **a**, **b** Transwell and wound-healing assays for KHOS cells after PD-L2 knockdown. **c**, **d** Transwell and wound-healing assays for U2OS cells after PD-L2 knockdown (magnification × 100). All experiments were repeated three times. Data are presented as the mean ± SD. **P < 0.01, ***P < 0.001

No significant differences were observed between the shPD-L2 and shNC groups in the cell colony-formation assay (Fig. 2d) or CCK-8 assay (Fig. 2e); the same results were also observed after KHOS cells transfected with PD-L2 overexpression vector (Additional File 2A). Both the KHOS-shPD-L2 (Fig. 3a, b) and U2OS-shPD-L2 groups (Fig. 3c, d) exhibited lower migratory and invasive capacities compared with control groups as determined by the transwell assay and wound-healing assay. The PD-L2 expression recovery increased the migration and invasion of shPD-L2-KHOS cells (Additional File 2A). Together, these data indicate that PD-L2 is important for migration and invasion of osteosarcoma cells.

### PD-L2 knockdown suppresses EMT in osteosarcoma cells

**Fig. 4:**
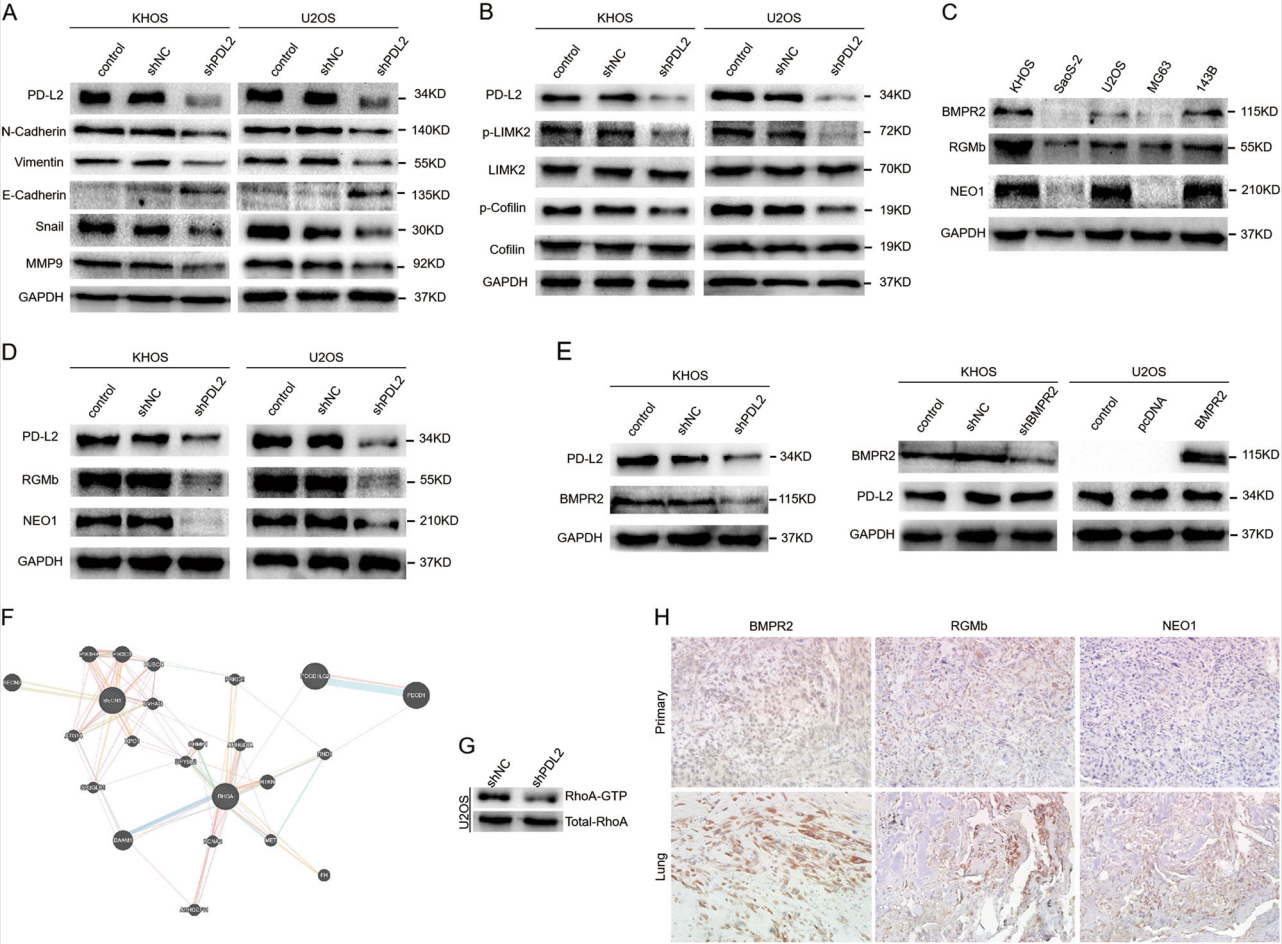
PD-L2 knockdown suppresses epithelial–mesenchymal transition and inactivates RhoA-ROCK-LIMK2 signaling. **a** EMT markers and MMP9 were measured by western blotting in KHOS and U2OS cells after transfection. **b** PD-L2 knockdown reduced p-LIMK and p-cofilin expression. **c** Expression of BMPR2, RGMb, and NEO1 in osteosarcoma cell lines detected by western blotting. **d** PD-L2 knockdown reduced RGMb and NEO1 expression. **e** PD-L2 knockdown decreased BMPR2 expression (top), but BMPR2 silencing and overexpression did not affect PD-L2 expression (bottom). **f** Bioinformatics prediction indicated there may be co-expression between PD-L2 and RhoA (http://genemania.org/). **g** GTPase assay verified RhoA changes in U2OS cells. **h** The expression levels of BBRN supercomplex (RGMb, BMPR2, and Neo1) in the primary and metastatic osteosarcoma tumors were evaluated by IHC (magnification × 200)

EMT is a critical process for epithelial cells to acquire a mesenchymal phenotype and is closely related to tumor metastasis^17^. Thus, we investigated by WB whether EMT-associated markers are affected by PD-L2 in KHOS and U2OS cells. As shown in Fig. 4a, the epithelial marker E-cadherin was increased, whereas mesenchymal markers, including Vimentin and N-cadherin, decreased after PD-L2 knockdown. Moreover, expressions of the transcription factors snail and MMP9 were also downregulated in the shPD-L2 group compared with controls. Conversely, the PD-L2 expression recovery increased MMP9 and snail levels, and induced EMT in shPD-L2-KHOS cells (Additional File 2B). The quantification of WB results was shown in Additional File 3A and 5A. These results indicate that PD-L2 knockdown inhibits EMT in osteosarcoma cells.

### PD-L2 knockdown inactivates RhoA-ROCK-LIMK2 signaling via BMPR2 and the RGMb/neogenin pathway

As a co-receptor, repulsive guidance molecule b (RGMb) integrates with bone morphogenetic protein/BMP receptor (BMP/BMPR) and neogenin (NEO1) to form a signaling supercomplex of BMP-BMPR-RGMb-neogenin (BBRN supercomplex)^18–21^. PD-L2 may interact with the BBRN supercomplex by binding to RGMb and regulate downstream signaling, including the RGMb-neogenin-RhoA pathway^18^. In our previous study^22^, we demonstrated that BMPR2 promoted invasion and metastasis via the RhoA-ROCK-LIMK2 pathway in osteosarcoma. In this study, PD-L2 knockdown decreased expression of p-LIMK2 and p-cofilin, but the corresponding total protein levels were unchanged in KHOS and U2OS cells (Fig. 4b). Furthermore, BMPR2, RGMb, and neogenin were detected in osteosarcoma cell lines by WB (Fig. 4c). Decreased expressions of RGMb and neogenin were observed in these two cell lines after PD-L2 silencing (Fig. 4d). As seen in Fig. 4c, KHOS cells exhibited higher levels of BMPR2 protein than U2OS cells, so we next investigated BMPR2 expression in KHOS cells after PD-L2 knockdown. As shown in Fig. 4e, BMPR2 expression was also inhibited by PD-L2 knockdown in KHOS cells. However, neither suppression of BMPR2 in KHOS nor overexpression of BMPR2 in U2OS affected PD-L2 expression (Fig. 4e). The PD-L2 expression recovery increased p-LIMK2, p-cofilin, RGMb, neogenin, and BMPR2 expressions in shPD-L2-KHOS cells (Additional File 2C). Furthermore, we detected the ROCK1 and ROCK2 expressions in osteosarcoma cell lines after PD-L2 knockdown. When PD-L2 was knocked down in KHOS and U2OS cells, there was no changes in expression of ROCK1, whereas ROCK2 expression was inhibited (Additional File 1B). In addition, bioinformatics prediction indicated that there may be co-expression between PD-L2 and RhoA (Fig. 4f), in agreement with the observed decreases in RhoA activation in PD-L2-knockdown U2OS cells compared with controls using a GTPase assay (Fig. 4g). The expression levels of BBRN supercomplex (RGMb, BMPR2, and Neo1) in the primary and metastatic osteosarcoma tumors were evaluated by IHC, and the results indicated BBRN supercomplex expression was increased in lung metastases compared with primary osteosarcoma (Fig. 4h). The quantification of WB results was shown in Additional File 3B-E and 5B.

Taken together, our data illustrate that PD-L2 activates RhoA-ROCK-LIMK2 signaling via BMPR2 and RGMb/neogenin pathways, and PD-L2 also stimulates LIMK2 through the small GTPase RhoA, resulting in downstream signaling changes.

### PD-L2 knockdown-induced attenuation of autophagy inhibits migration and invasion of osteosarcoma cells that occurs via RhoA-ROCK-LIMK2 signaling

**Fig. 5:**
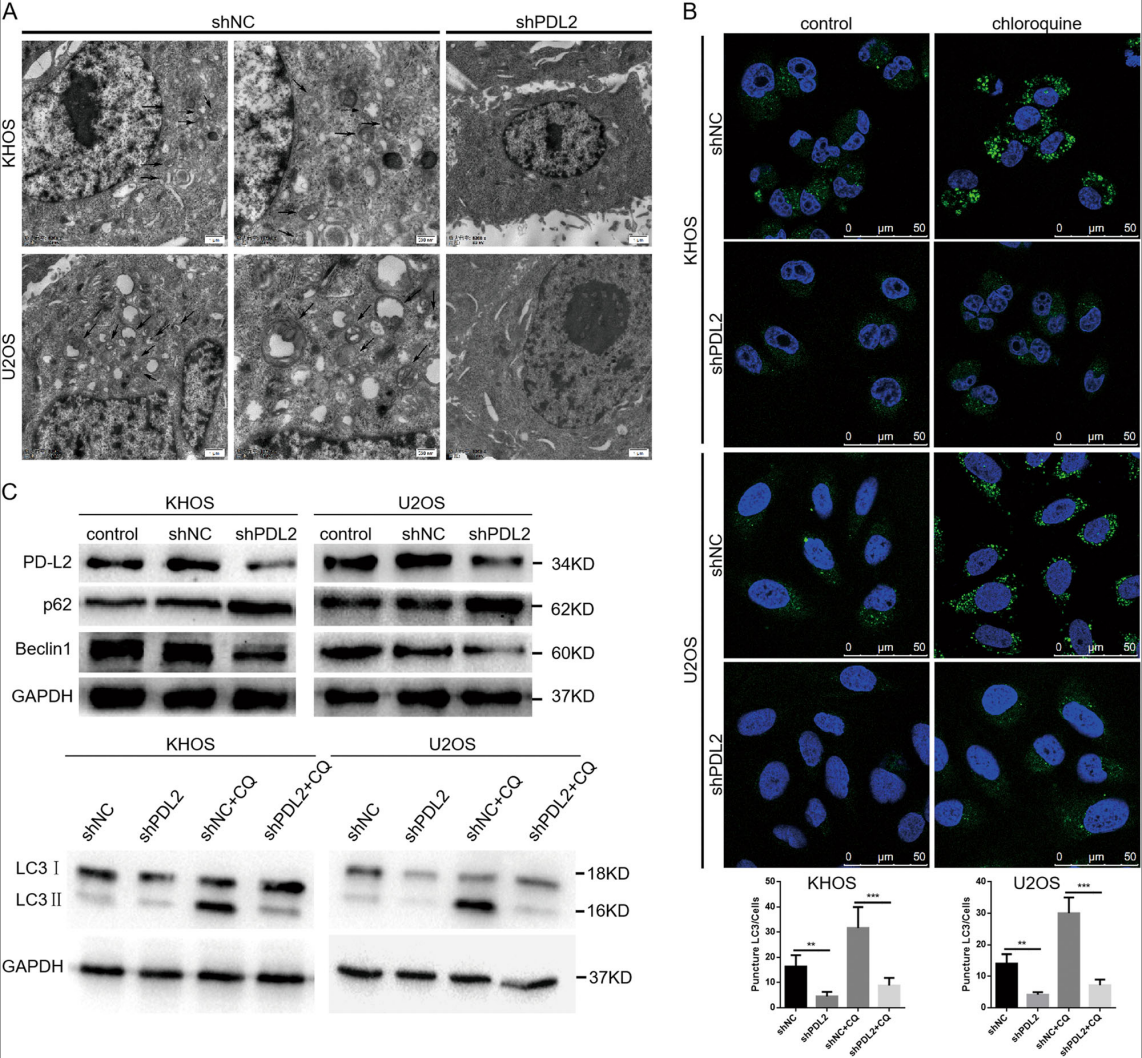
PD-L2 knockdown-induced inhibition of autophagy attenuates migration and invasion of osteosarcoma cells. **a** Representative TEM images depict ultrastructures present during autophagy in KHOS and U2OS cells transfected with PD-L2 shRNA or shNC. Images show autophagic vacuoles (arrows) observed in control cells (the picture in the right is a zoom of the picture in the left). No or few autophagic vacuoles were observed in PD-L2 knockdown cells. **b** Cells after PD-L2 knockdown exhibited a punctate pattern of LC3-II fluorescence, with reduced LC3-II compared with autophagosomes. KHOS and U2OS cells were incubated with or without CQ. **c** Expression of LC3, p62, and beclin-1 was evaluated by western blot. KHOS and U2OS cells were incubated in the presence or absence of CQ

To determine whether PD-L2 is involved in autophagy, transmission electron microscopy (TEM) was used to detect ultrastructures during autophagy. PD-L2 silencing induced few autophagic vesicles compared with the control groups in KHOS and U2OS cells (Fig. 5a). In addition, to measure LC3 conversion from LC3-I to LC3-II during autophagy, immunofluorescence was used to detect LC3-II expression in autophagosomes in osteosarcoma cells. Our results illustrate that LC3-II expression was decreased after PD-L2 knockdown in osteosarcoma cells (Fig. 5b). In addition, decreased LC3-II and beclin-1 levels accompanied by increased p62 expression were observed by WB after PD-L2 knockdown in osteosarcoma cells (Fig. 5c). In addition, when combined with chloroquine (CQ), an inhibitor of the degradation step of autophagy, the expression of LC3-II enhanced when compared with PD-L2 knockdown alone (Fig. 5b, c). The PD-L2 expression recovery increased LC3-II and beclin-1 levels, and decreased p62 expression in shPD-L2-KHOS cells (Additional File 2D), which indicated PD-L2 promoted autophagy (Additional File 2E). The quantification of WB results was shown in Additional File 4A and 5C.

**Fig. 6:**
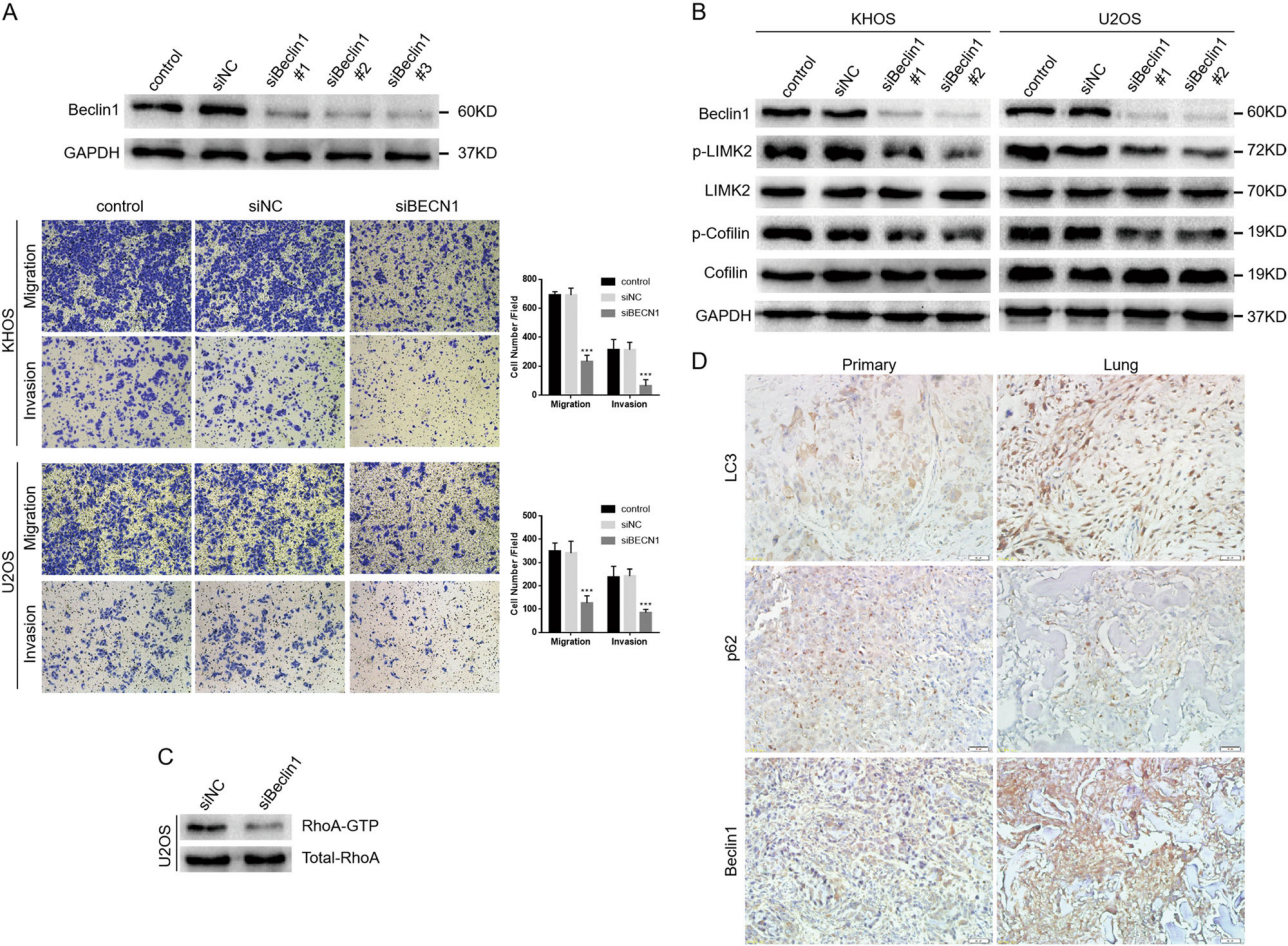
Autophagy promotes migration and invasion of osteosarcoma cells through targeting the RhoA-ROCK-LIMK2 pathway. (**a**) Three beclin-1 siRNA sequences were used to downregulate beclin-1 in KHOS cells (top). The migration and invasion of osteosarcoma cells after beclin-1 knockdown were analyzed by transwell and wound-healing assays (bottom). (**b** and **c**) Bioinformatics prediction indicated there may be co-expression between beclin-1 and RhoA, and western blot validated the relationship between them. Beclin-1 knockdown decreased p-LIMK and p-cofilin expressions (**b**) as well as RhoA activation (**c**). **d** The expression levels of autophagy markers (LC3, p62, Beclin1) in the primary and metastatic osteosarcoma tumors were evaluated by IHC (magnification 200X). All experiments were repeated three times. Data are presented as the mean ± S.D. ***P < 0.001

Autophagy may either inhibit or enhance migration and invasion of tumor cells, depending on the cellular context^23,24^. Considering that autophagy regulation may increase the efficacy of anticancer therapeutics, we next investigated whether the autophagy inhibition induced by PD-L2 knockdown affects migration and invasion of osteosarcoma cells. We transfected the small interfering RNA (siRNA) targeting beclin-1 into KHOS and U2OS cells to inhibit autophagy, and decreased LC3-II and increased p62 expression were observed by WB (Additional File 1C). Transwell assay experiments revealed beclin-1 silencing significantly attenuated migratory and invasive abilities of KHOS and U2OS cells (Fig. 6a). The quantification of WB results was shown in Additional File 4B.

Furthermore, the bioinformatics prediction also indicated that there may be co-expression between beclin-1 and RhoA, and the above results support the relationship between PD-L2 and RhoA. Therefore, we next investigated the effect of beclin-1 on RhoA and its downstream signals. In our study, reduced expression of p-LIMK2 and p-cofilin were detected by WB after beclin-1 silencing, and beclin-1 knockdown also resulted in decreased RhoA activation (Fig. 6b, c). The expression levels of autophagy markers including LC3, p62, and Beclin1 in the primary and metastatic osteosarcoma tumors were evaluated by IHC, and the results indicated autophagy was more likely to occur in lung metastases compared with primary osteosarcoma (Fig. 6d). The quantification of WB results was shown in Additional File 4C-D. These data suggest that autophagy promotes migration and invasion of osteosarcoma cells through targeting the RhoA-ROCK-LIMK2 pathway.

### PD-L2 knockdown inhibits osteosarcoma cell metastasis in vivo

**Fig. 7:**
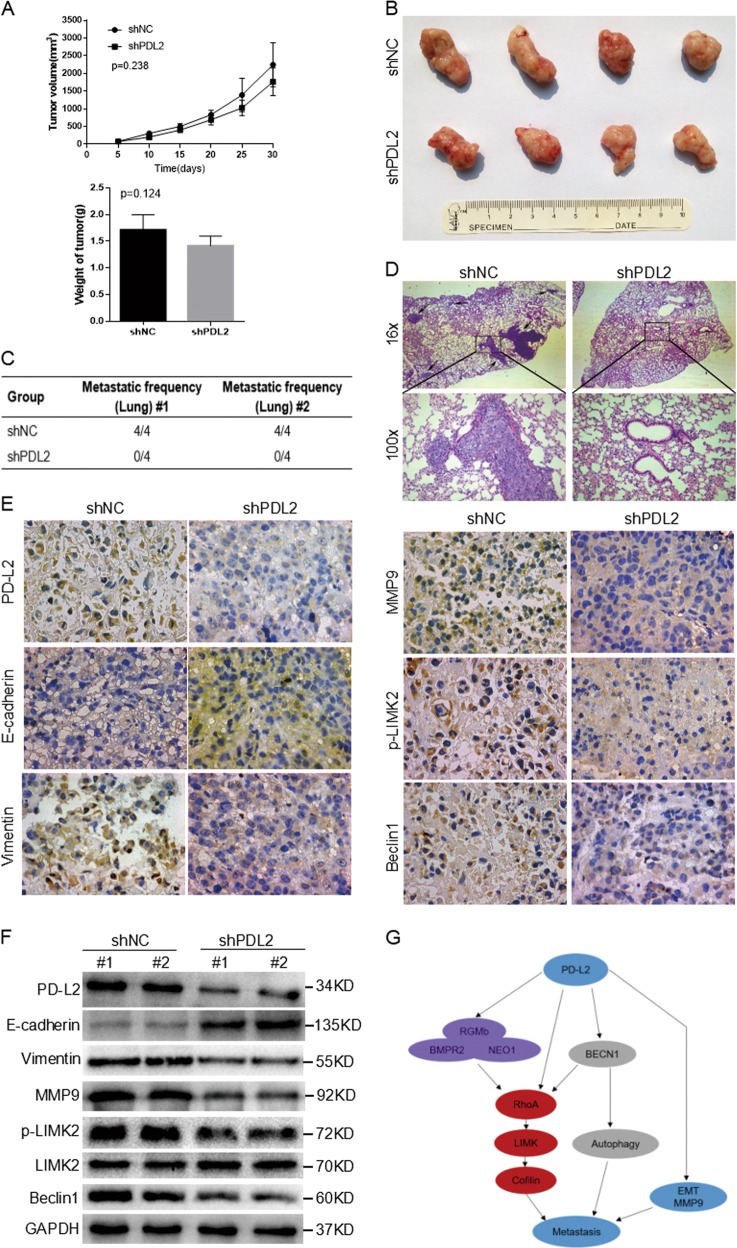
PD-L2 knockdown inhibits osteosarcoma cells metastasis in vivo. **a** Tumor growth curves and tumor weights were evaluated between shPD-L2 and shNC groups. No significant difference was observed between the two groups. **b** Representative images of primary tumors. **c** The metastatic frequency of shNC and shPD-L2 groups. **d** H&E staining of the lungs of shNC and shPD-L2 groups (magnification × 16 and × 100). Arrows indicate metastases. **e**, **f** Primary tumors were collected and evaluated by IHC (magnification × 400) and western blotting. **g** A diagrammatic sketch of PD-L2 in osteosarcoma metastasis. Data are presented as the mean ± SD (*n* = 4)

To validate our in vitro results, we determined whether PD-L2 knockdown influences growth and spontaneous metastasis of osteosarcoma cells in vivo. No significant differences were observed in tumor volume or weight between shPD-L2 and shNC groups (Fig. 7a, b); however, lung metastases were found in all four mice (4/4) from the shNC group, whereas no lung metastatic nodes were found in the shPD-L2 group (0/4) (Fig. 7c). Statistical differences were observed between these two groups using Student’s *t*-test. Representative hematoxylin and eosin (H&E)-stained images of lungs are shown (Fig. 7d).

Primary tumors were collected and evaluated by IHC and WB. In accordance with in vitro findings, increased E-cadherin expression with decreased vimentin, MMP9, p-LIMK2, and beclin-1 expression were detected in tumors from the shPD-L2 group compared with the shNC group (Fig. 7e, f). The quantification of WB results was shown in Additional File 4E.

In conclusion, PD-L2 knockdown inhibits metastasis in osteosarcoma cells in vivo.

## Discussion

As homologous ligands for PD-1, both PD-L1 and PD-L2 negatively regulate the activation of T-cells. The existing study examined the expression levels of PD-L1, PD-L2, and PD-1 in multiple sarcomas including osteosarcoma and the PD-1 axis effectors are differentially expressed in various sarcomas varying from 0 to 40%^16^. Moreover, the results indicated osteosarcoma, which is the most common primary malignant bone tumor with a high mortality and disability rate, exhibits relatively high expression levels of PD-1 axis effectors. The current study indicates that metastatic, but not primary, osteosarcoma tumors express PD-L1 and PD-1 with ~75% positivity rate^13,14^, whereas some studies exhibit that PD-L1 is detected in primary osteosarcoma with positivity rates of 6.8% and 35.5%, respectively^15,16^, and no significant differences are observed between primary and metastatic osteosarcoma. As for PD-L2, the positivity rates of PD-L2 in osteosarcoma is 41.9%^16^. Previous studies have shown that PD-L1 and PD-L2 have different prognostic roles in various tumors^25–31^. The consistencies and discrepancies in the prognostic effects of PD-L1 and PD-L2 may be due to their overlapping immune escape function and varying tumor cell-intrinsic molecular functions; however, there are few studies that address this subject. Of note, PD-L2 is reported to be significantly elevated in “mesenchymal” lung adenocarcinoma^11^. In our study, we show that PD-L2 expression is higher in metastatic osteosarcoma compared with primary osteosarcoma by IHC, and that PD-L2 knockdown inhibits migration and invasion of KHOS and U2OS cells. Next, we further investigated possible molecular mechanisms for these observations.

EMT is well known to be involved in tumor metastasis^32–34^. The epithelial phenotype is detected in some sarcomas, including osteosarcoma^35^, whereas a MET-like phenomenon was observed in chondrosarcoma after reducing snail^36^. In our study, PD-L2 knockdown inhibits EMT in KHOS and U2OS cells, resulting in increased E-cadherin and decreased N-cadherin, vimentin, snail, and MMP9, both in vitro and in vivo.

RGMb, a secondary receptor for PD-L2^18^, is a member of the repulsive guidance molecule (RGM) family that includes RGMa, RGMb, and RGMc^37^, and is expressed in the nervous system, macrophages, and other cells of the immune system^37,38^. Moreover, RGMb is also reported to be expressed in some tumors and has varying roles^39–41^. Previous studies show that RGMb is associated with tumorigenesis, immunoregulation, and cell-to-cell adhesion, and has an important role in the RGMb-neogenin-RhoA signaling pathway^19,42–45^. As a co-receptor, RGMb binds BMPs and neogenin^19,20^, whereas neogenin also directly binds BMP-2/4/6/7^46,47^. In general, RGMb coordinates with diverse receptors to form a BBRN supercomplex and PD-L2 may regulate downstream signaling through interacting with this BBRN supercomplex^18^.

Previous studies have shown that regulation of the actin cytoskeleton has a vital role in cell migration, a process in which LIMK and cofilin are crucial regulators^48,49^. Phosphorylation of cofilin inhibits its binding to actin filaments, which restrains filament disassembly. Phosphorylation of cofilin is regulated by LIMK, a downstream molecule of RhoA. In our previous study^22^, we demonstrated that BMPR2 knockdown inhibits phosphorylation of LIMK2 and cofilin, resulting in reducing cell invasion and lamellipodia extension. BMPR2 also increases RhoA activation and interacts with LIMK2 directly. In our study, decreased phosphorylation of LIMK2 and cofilin were observed after PD-L2 knockdown in KHOS and U2OS cells. Furthermore, PD-L2 knockdown also inhibited expression of RGMb, neogenin, and BMPR2, indicating that PD-L2 is upstream of these molecules and positively regulates expression of BBRN supercomplex signaling. In fact, PD-L2, RGMb, neogenin, BMP, and its receptors constitute a complicated supercomplex cell signaling network, whereas the function and regulation of the cross-talk between these components remains to be further elucidated. Some studies have shown the expression and interaction of RGMb/neogenin, RGMb/BMPs, or neogenin/BMPs, and most of the researches on RGMb/neogenin mainly focus on the nervous system^19–21,46,47^. RGMb is also reported to be expressed in some tumors such as breast cancer, colorectal cancer, and lung cancer, and has varying roles;^39–41^ abnormal expression of neogenin has been found in pancreatic carcinoma, colon cancer, esophageal squamous cell carcinoma, gliomas, and breast cancer;^50^ BMP and its receptors are also implicated with many cancers^22^. However, the systematic study for BBRN expression in tumor is rare.

It is well known that autophagy regulates apoptosis, but how autophagy influences metastasis remains unknown. As a double-edged sword, autophagy may either inhibit or enhance tumor cell metastasis, depending on the cellular context and stimulus^51^. For osteosarcoma, the similar phenomenon is also observed that autophagy could either promote or suppress metastasis under the background of different gene regulation^52–54^. Recent studies demonstrate that autophagy and EMT are linked in a complex relationship, and autophagy could induce or restrain EMT in different situations^55,56^. Furthermore, some evidence indicates the cytoskeleton may be one of the crucial regulatory roles at the crossroad between these two biological processes^57^. Complicated regulation has been observed between autophagy and the RHO family or actin cytoskeleton in previous studies^58^. Currently, literature describing the relationship between PD-L2 and autophagy is scarce. In this study, we demonstrate that PD-L2 knockdown-mediated inhibition of autophagy is an anti-metastatic mechanism that suppresses migration and invasion via inactivation of RhoA-ROCK-LIMK2 signaling.

Our data illustrate that PD-L2 activates the RhoA-ROCK-LIMK2 pathway through the BBRN supercomplex and its direct effect on RhoA activation; however, PD-L2 induces autophagy via beclin-1, whereas beclin-1 can also independently activate RhoA-ROCK-LIMK2 pathway, resulting in autophagic regulation of cell migration and invasion. Through the effect of these two pathways, PD-L2 activates the RhoA-ROCK-LIMK2 pathway to affect cell adhesion and motility (Fig. 7g).

In summary, our study reveals for the first time that PD-L2 functions as a pro-metastatic oncogene and regulates autophagy. PD-L2 knockdown inhibits metastasis through the RhoA-ROCK-LIMK2 and autophagy pathways, which extends our comprehension of the regulatory function of autophagy in tumor cell metastasis and represents a potential therapeutic target in osteosarcoma.

## Materials and methods

### Osteosarcoma tissue samples and patient information

Formalin-fixed and paraffin-embedded primary osteosarcoma and matched pulmonary metastasis specimens from 18 patients were acquired from the musculoskeletal tumor center of Peking University People’s Hospital. Informed consent was obtained from all patients and this study was approved by the ethics committee of Peking University People’s Hospital.

### Cell culture and reagents

KHOS, 143B, SAOS-2, U2OS, and MG63 cells were obtained from American Type Culture Collection (Manassas, VA, USA). KHOS, SAOS-2, U2OS, and MG63 cells were recently authenticated in Beijing Microread Genetics, Co., Ltd by Short Tandem Repeat (STR) analysis. 143B cells were recently authenticated in Cobioer Biosciences, Co., Ltd by STR analysis. KHOS and U2OS cells were cultured in RPMI 1640 medium (Hyclone, Logan, UT, USA). 143B, SAOS-2, and MG63 cells were maintained in Dulbecco’s modified Eagle’s medium (Hyclone, Logan, UT, USA). Cell culture medium was supplemented with 10% fetal bovine serum (Gibco) and 1% penicillin/streptomycin (Invitrogen). All cell lines were cultured at 37 °C with 5% CO_2_. CQ was obtained from Selleck (Houston, TX, USA).

### Flow cytometry

All osteosarcoma cell lines were analyzed for PD-L2 expression by flow cytometry. The cells were prepared and incubated with the primary antibody for 30 min at 4 °C and then washed with phosphate-buffered saline (PBS) according to the manufacturer’s instructions. After washing, cells were assayed using an Accuri C6 flow cytometer (BD Biosciences, San Diego, CA, USA). Fluorescent antibodies including APC-PD-L2 (17-5888) and the isotype control (17-4714) were purchased from eBioscience.

### IHC and immunofluorescence assays

IHC staining was performed as described previously^59^. Assessment of immunostaining was conducted by two independent pathologists without any previous knowledge of the clinical specimens. The score for positive IHC staining percentage was grouped into four levels: 0: 0% positive; 1: < 5% positive; 2: 5–50% positive; and 3: > 50% positive. The score for staining intensity was also grouped into four levels: 0: none; 1: weak; 2: moderate; and 3: intense. Total score = percentage score × intensity score. Based on the scoring system, more than ten representative fields (× 400 magnification) were calculated for IHC analysis between primary osteosarcoma and lung metastasis groups. Antibodies for IHC against PD-L2 (82723)^60^, E-cadherin (3195), and Vimentin (5741) were purchased from Cell Signaling Technology (CST, Beverly, MA, USA). Anti-MMP9 (ab76003) and p-LIMK2 (ab38499) were purchased from Abcam (Cambridge, UK), and anti-Beclin1 (11306-1-AP) was purchased from Proteintech Group, Inc. (Chicago, USA).

For the immunofluorescence assay of LC3, cells were fixed and permeabilized with 0.1% Triton X-100 at room temperature for 15 min and then incubated with anti-LC3 antibody overnight at 4 °C. Cells were washed three times with PBS and incubated with a Cy3-conjugated goat anti-rabbit IgG secondary antibody for 1 h at room temperature. Cells were viewed and imaged using confocal microscopy (FV10i, Olympus, Tokyo, Japan).

### WB and GTPase assay

WB was performed as previously described^59^. Anti-human BMPR2 (ab78422), p-LIMK2 (ab38499), LIMK2 (ab97766), MMP9 (ab76003), PD-L2 (ab187662), and the RhoA activation assay (ab211164) were purchased from Abcam (Cambridge, UK). Antibodies against E-cadherin (3195), N-cadherin (13116), Vimentin (5741), Snail (3879), p-cofilin (3313), cofilin (5175), ROCK1 (4035), ROCK2 (9029), LC3 (12741), p62 (8025), and Beclin-1 (3495) were purchased from CST (Beverly, MA, USA). Anti-GAPDH (sc-25778) and PD-L1 (sc-50298) were purchased from Santa Cruz Biotechnology (Santa Cruz, CA, USA). The GTPase assay was conducted according to the manufacturer’s instructions.

### Quantitative reverse-transcription PCR

Total RNA was isolated using Trizol (Invitrogen) and cDNAs were synthesized with purified RNA and OligdT primers using SuperScript III First-Strand Synthesis SuperMix (Invitrogen). Real-time quantitative PCR was performed using the SYBR-Green PCR Master Mix (Applied Biosystems, Foster City, CA, USA) on Bio-Rad CFX96 (Applied Biosystems, CA, USA). Relative transcript expression was normalized to GAPDH. All protocols were conducted as per the manufacturer’s instructions.

The primer sequences were as follows: PD-L2 forward 5′- AAAGAGCCACTTTGCTGGAG-3′ and PD-L2 reverse 5′- GAGGACGTAGTAACGAAAGT-3′; GAPDH forward 5′-GCACCGTCAAGGCTGAGAAC-3′ and GAPDH reverse 5′-ATGGTGGTGAAGACGCCAGT-3′.

### Gene knockdown with siRNA/shRNA and overexpression with adenovirus

Lentiviruses targeting PD-L2 and BMPR2 were obtained from GenePharma (Suzhou, China). A non-targeting lentivirus construct was used as a negative control (NC). The shRNA sequences were as follows: PD-L2 pair 1, sense strand 5′-CCTAAGGAACTGTACATAA-3′ and antisense strand 5′-TTATGTACAGTTCCTTAGG-3’; PD-L2 pair 2, sense strand 5′-CCATCCAACTTGGCTGCTT-3′ and antisense strand 5′-AAGCAGCCAAGTTGGATGG-3′; BMPR2 pair 1, sense strand 5′-GCCTATGGAGTGAAATTATTT-3′ and antisense strand 5′-AAATAATTTCACTCCATAGGC-3′; BMPR2 pair 2, sense strand 5′-CCTAACTGTATACCAGAATTA-3′ and antisense strand 5′-TAATTCTGGTATACAGTTAGG-3’; and NC, sense strand 5′-TTCTCCGAACGTGTCACGT-3′ and antisense strand 5′-ACGTGACACGTTCGGAGAA-3′. ShPD-L2, shBMPR2, and NC stably expressing osteosarcoma cells were generated using puromycin (2 μg/ml) for 2 weeks after infection with lentivirus. Overexpression of BMPR2 was performed using an adenoviral vector (Vigene, Shanghai, China) carrying the full human genomic BMPR2 DNA (NM_001204) fused with enhanced green fluorescent protein (EGFP); an empty adenoviral vector that only contained EGFP was used as a NC. Transfections of lentivirus and adenovirus were conducted according to the manufacturer’s instructions.

SiBeclin-1 and nonspecific NC siRNA (siNC) were purchased from GenePharma (Suzhou, China). The sequences targeting Beclin-1 were as follows: #1, 5′-CAAGUUCAUGCUGACGAAUTT-3′ (sense) and 5′-AUUCGUCAGCAUGAACUUGAG-3′ (antisense); #2, 5′-CAGGAUGAUGUCCACAGAATT-3′ (sense) and 5′-UUCUGUGGACAUCAUCCUGGC-3′ (antisense); #3, 5′-CGUGGAAUGGAAUGAGAUUTT-3′ and (sense) and 5′-AAUCUCAUUCCAUUCCACGGG-3′ (antisense). The nonspecific siRNAs used as control were as follows: 5′-UUCUCCGAACGUGUCACGUTT-3′ (sense) and 5′-ACGUGACACGUUCGGAGAATT-3′ (antisense). The PD-L2 overexpression plasmid and NC were purchased from GenePharma (Suzhou, China). siRNAs and plasmid were transfected into tumor cells using Lipofectamine 3000 (Invitrogen, Carlsbad, CA, USA) according to the manufacturer’s instructions. After 48 h transfection, cells were collected for further analyses.

### Transmission electron microscopy

For TEM, cells were digested with 0.25% trypsin, suspended at a concentration of 1.0 × 10^6^ per ml and fixed with 1.5% glutaraldehyde at 4 °C for 6 h. Ultrathin sections (100 nm) were prepared, stained with uranyl acetate and lead citrate, and examined under a TEM (H-600; Hitachi, Tokyo, Japan).

### CCK-8 assay

The CCK-8 (Dojindo Laboratories, Kumamoto, Japan) assay was used to examine the cell viability according to the manufacturer’s instruction. Cells were plated in 96-well plates at a density of 5000 cells in 100 μl medium per well 1 day before the experiment.

### Cell colony-formation assay

Cells (1 ×10^3^) in the logarithmic phase were plated into six-well plates, allowed to form colonies for 10 days, and then fixed with methyl alcohol and stained with crystal violet staining solution.

### Wound-healing assay

Cells (4 × 10^5^) were seeded onto a six-well plate, after which confluent cells were scratched using 200 µl sterile pipette tips in each well. Images of wounded areas were taken at 0 and 24 h (KHOS) or 0 and 36 h (U2OS). All images were analyzed using Image J software (Rawak Software, Inc., Germany).

### Transwell assay

Cells (6 × 10^4^) were seeded into the non-coated upper chamber of transwell plates (8 µm pore size; Corning) for a migration assay and into Matrigel-coated upper chamber (BD Bioscience, 354234) for an invasion assay. After culturing for 24 h, cells were fixed with methanol and stained with a 0.1% crystal violet solution. Migrated cell populations were evaluated in five fields per well under a microscope.

### Tumor xenografts

To evaluate the effect of PD-L2 knockdown on primary tumor growth and spontaneous metastasis, 6-week-old female BALB/c nude mice (Vitalriver, Beijing, China) were orthotopically injected in the para-osseous proximal tibia with 5 × 10^6^ KHOS-shPD-L2 or -shNC cells. Tumor volume was measured beginning 5 days post injection as (length × width^2^)/2 and measured every 5 days thereafter until killing at 30 days. Upon termination of the study, tumors were collected, weighed, photographed, and processed for WB and IHC assays. Lungs were processed for routine H&E staining and the number of metastatic nodules in the lung was quantified.

### Statistical analysis

All statistical analyses were performed using SPSS v.21.0 software (SPSS, Chicago, IL, USA). Statistical evaluation was performed using Student’s *t*-test. Data are expressed as the mean ± SD. In all statistical analyses, a *P*-value < 0.05 was considered statistically significant in a two-sided test.

## Supplementary information

Expression of PD-L1, ROCK1 and ROCK2 after PD-L2 knockdown and expression of LC3, p62 after Beclin-1 knockdown in osteosarcoma cells

Effect of PD-L2 expression recovery on proliferation, migration, invasion and autophagy of osteosarcoma cells

The quantification of western blot results in Figure 4

The quantification of western blot results in Figure 5 and Figure 6

The quantification of western blot results in Figure S1 and S2

Supplementary figure legends
