# Supplementary material for: Osteosarcoma cell intrinsic PD-L2 signals promote invasion and metastasis via the RhoA-ROCK-LIMK2 and autophagy pathways
Source: Cell Death Dis. 2019 Mar 18;10(4):261. doi: 10.1038/s41419-019-1497-1 (PMC6423010; doi:10.1038/s41419-019-1497-1)
Supplement: Supplementary file 1 — Expression of PD-L1, ROCK1 and ROCK2 after PD-L2 knockdown and expression of LC3, p62 after Beclin-1 knockdown in osteosarcoma cells [file 41419_2019_1497_MOESM1_ESM.doc]

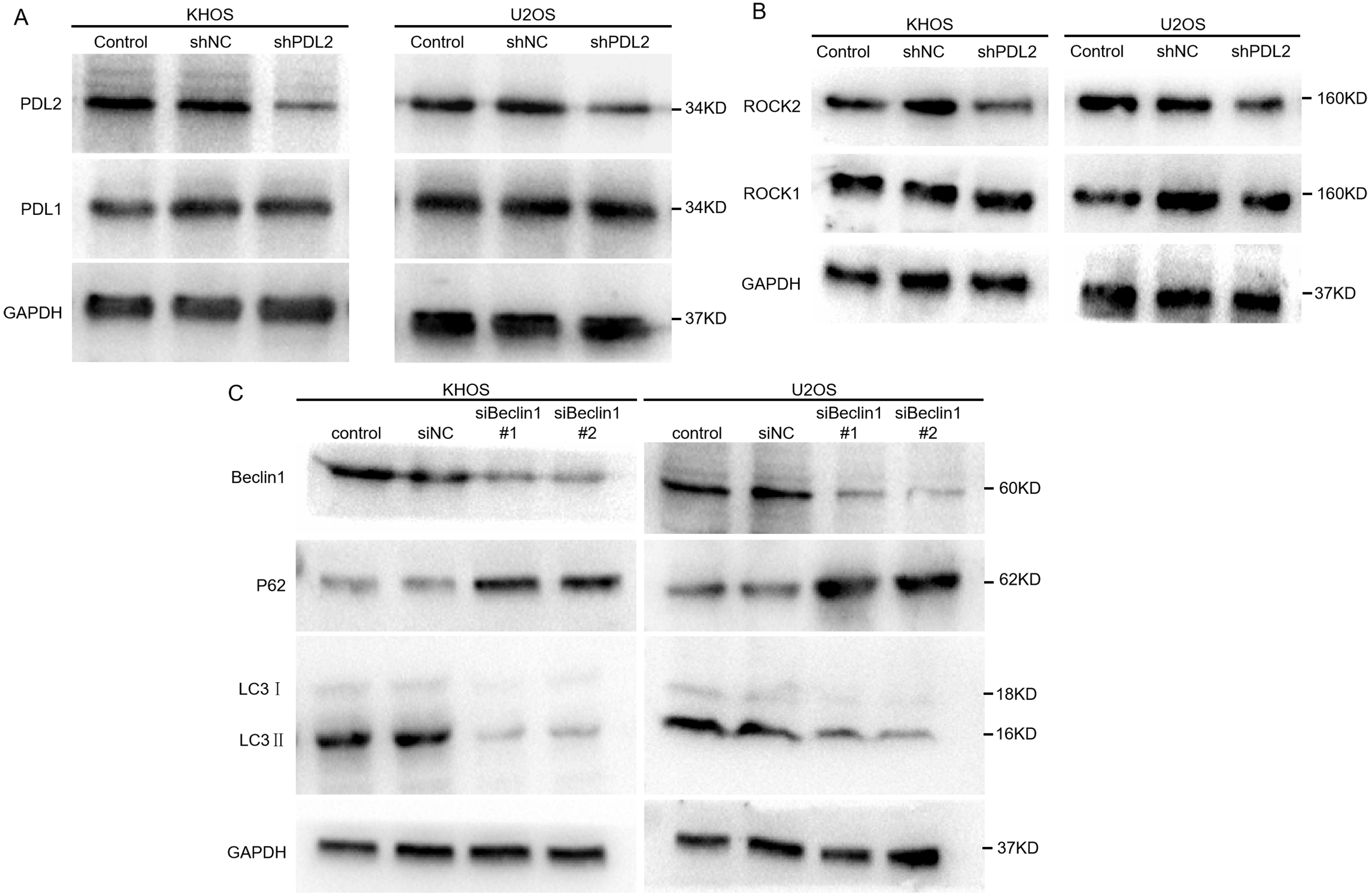


Figure S1: Expression of PD-L1, ROCK1 and ROCK2 after PD-L2 knockdown and expression of LC3, p62 after Beclin-1 knockdown in osteosarcoma cells. (A)The expression of PD-L1 remained no changes after PD-L2 knockdown in KHOS and U2OS cells, which was detected by western blot. (B) ROCK2 expression was inhibited after PD-L2 knockdown in KHOS and U2OS cells (C) Western blot analysis was used to evaluate the expression of LC3 and p62 after beclin1 knockdown which markedly decreased autophagy.
