# Supplementary material for: Osteosarcoma cell intrinsic PD-L2 signals promote invasion and metastasis via the RhoA-ROCK-LIMK2 and autophagy pathways
Source: Cell Death Dis. 2019 Mar 18;10(4):261. doi: 10.1038/s41419-019-1497-1 (PMC6423010; doi:10.1038/s41419-019-1497-1)
Supplement: Supplementary file 2 — Effect of PD-L2 expression recovery on proliferation, migration, invasion and autophagy of osteosarcoma cells [file 41419_2019_1497_MOESM2_ESM.doc]

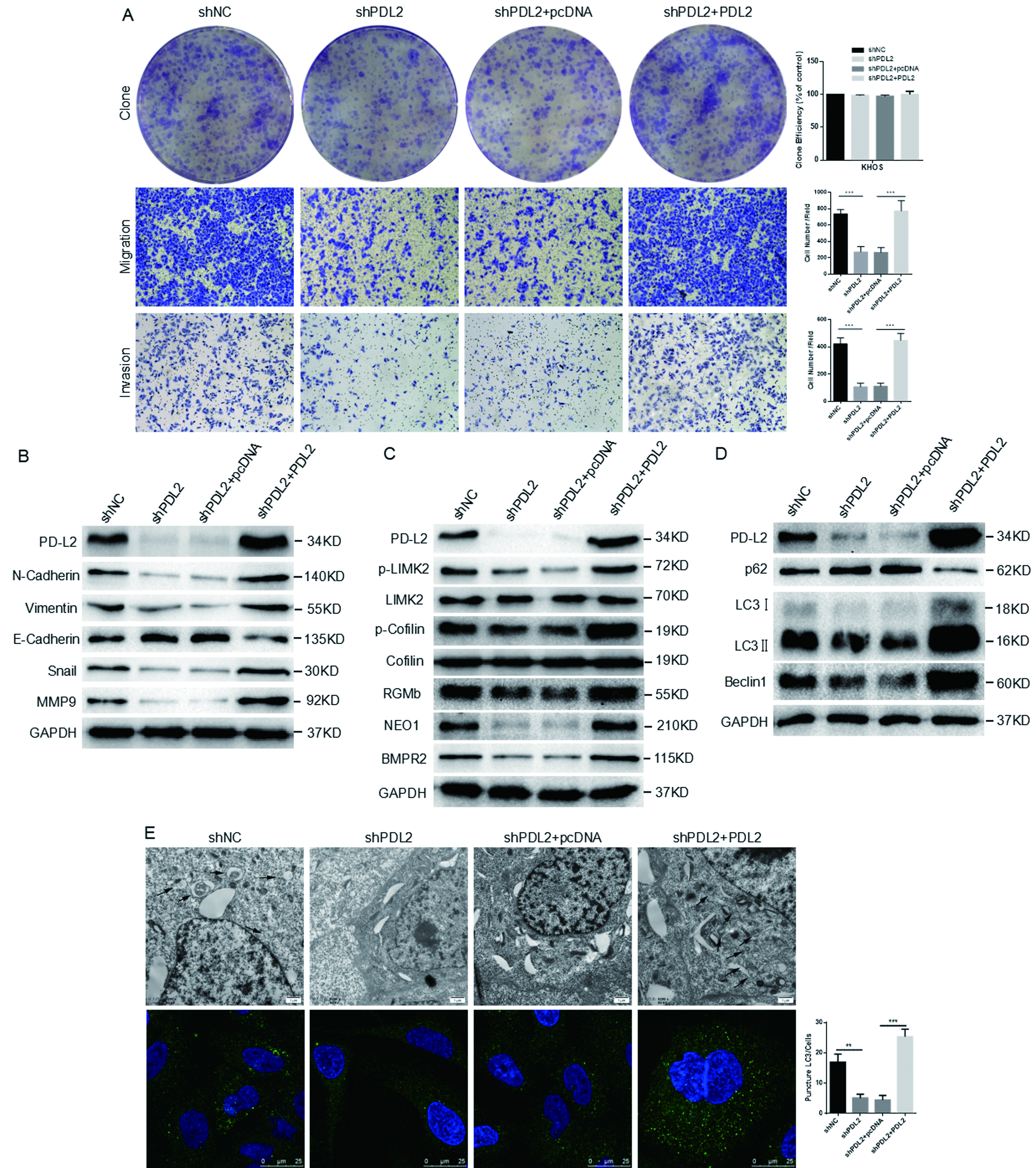


Figure S2: Effect of PD-L2 expression recovery on proliferation, migration, invasion and autophagy of osteosarcoma cells. (A) Cell proliferation, migration and invasion of KHOS cells after PD-L2 expression recovery were determined by cell colony formation and transwell assays. (B) The PD-L2 expression recovery increased MMP-9 and snail levels and induced EMT in shPDL2-KHOS cells. (C) The PD-L2 expression recovery increased p-LIMK2, p-cofilin, RGMb, neogenin and BMPR2 expressions in shPDL2-KHOS cells. (D) Western blot analysis was used to evaluate the expression of LC3, beclin1 and p62 after PD-L2 expression recovery. (E) Expression recovery of PD-L2 markedly increased autophagy. Data are presented as the mean ± S.D. **P<0.01, ***P<0.001.
