# Supplementary material for: Osteosarcoma cell intrinsic PD-L2 signals promote invasion and metastasis via the RhoA-ROCK-LIMK2 and autophagy pathways
Source: Cell Death Dis. 2019 Mar 18;10(4):261. doi: 10.1038/s41419-019-1497-1 (PMC6423010; doi:10.1038/s41419-019-1497-1)
Supplement: Supplementary file 3 — The quantification of western blot results in Figure 4 [file 41419_2019_1497_MOESM3_ESM.doc]

Figure S3: The quantification of western blot results in Figure 4. (A) The quantification of western blot results in Figure 4A. (B) The quantification of western blot results in Figure 4B. (C) The quantification of western blot results in Figure 4D. (D) The quantification of western blot results in Figure 4E. (E) The quantification of western blot results in Figure 4G. Data are presented as the mean ± S.D. **P<0.01, ***P<0.001.
