# Supplementary material for: Osteosarcoma cell intrinsic PD-L2 signals promote invasion and metastasis via the RhoA-ROCK-LIMK2 and autophagy pathways
Source: Cell Death Dis. 2019 Mar 18;10(4):261. doi: 10.1038/s41419-019-1497-1 (PMC6423010; doi:10.1038/s41419-019-1497-1)
Supplement: Supplementary file 4 — The quantification of western blot results in Figure 5 and Figure 6 [file 41419_2019_1497_MOESM4_ESM.doc]

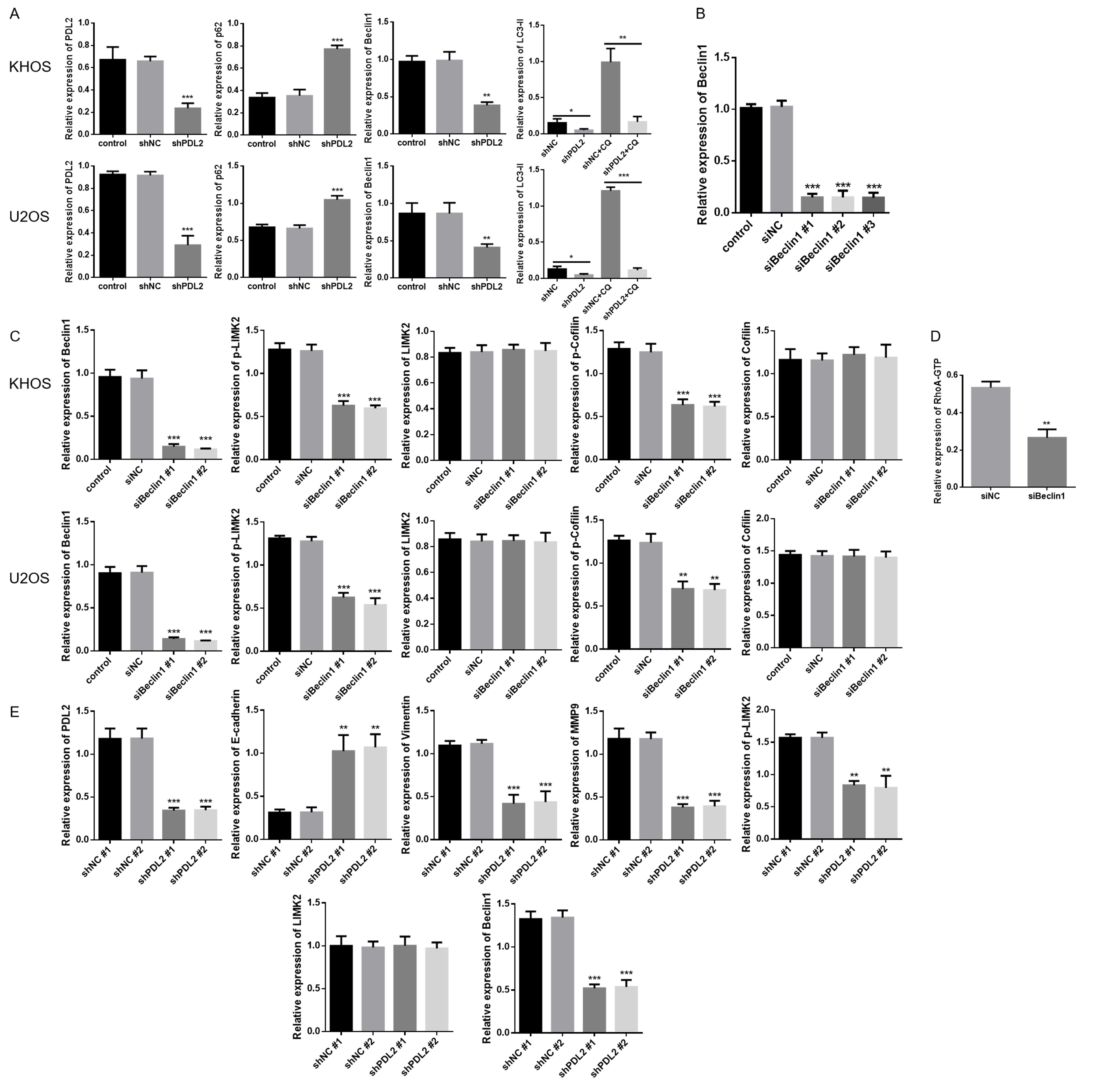


Figure S4: The quantification of western blot results in Figure 5, 6 and 7. (A) The quantification of western blot results in Figure 5C. (B) The quantification of western blot results in Figure 6A. (C) The quantification of western blot results in Figure 6B. (D) The quantification of western blot results in Figure 6C. (E) The quantification of western blot results in Figure 7F. Data are presented as the mean ± S.D. **P<0.01, ***P<0.001.
