# Supplementary material for: Osteosarcoma cell intrinsic PD-L2 signals promote invasion and metastasis via the RhoA-ROCK-LIMK2 and autophagy pathways
Source: Cell Death Dis. 2019 Mar 18;10(4):261. doi: 10.1038/s41419-019-1497-1 (PMC6423010; doi:10.1038/s41419-019-1497-1)
Supplement: Supplementary file 5 — The quantification of western blot results in Figure S1 and S2 [file 41419_2019_1497_MOESM5_ESM.doc]

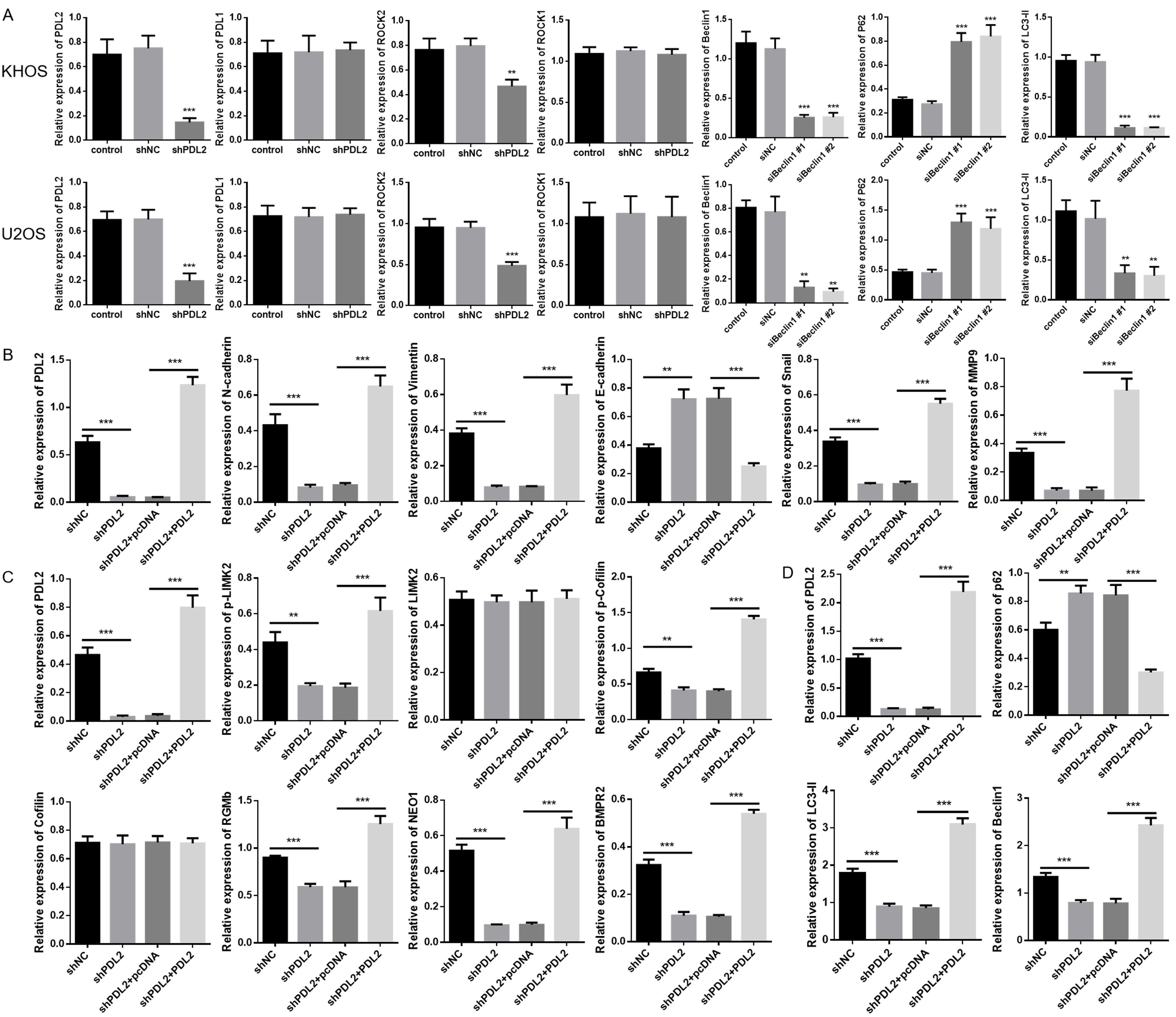


Figure S5: The quantification of western blot results in Figure S1 and S2. (A) The quantification of western blot results in Figure S1. (B) The quantification of western blot results in Figure S2B. (C) The quantification of western blot results in Figure S2C. (D) The quantification of western blot results in Figure S2D. Data are presented as the mean ± S.D. **P<0.01, ***P<0.001.
