# Supplementary material for: Osteosarcoma cell intrinsic PD-L2 signals promote invasion and metastasis via the RhoA-ROCK-LIMK2 and autophagy pathways
Source: Cell Death Dis. 2019 Mar 18;10(4):261. doi: 10.1038/s41419-019-1497-1 (PMC6423010; doi:10.1038/s41419-019-1497-1)
Supplement: Supplementary file 6 — Supplementary figure legends [file 41419_2019_1497_MOESM6_ESM.doc]

Figure S1: Expression of PD-L1, ROCK1 and ROCK2 after PD-L2 knockdown and expression of LC3, p62 after Beclin-1 knockdown in osteosarcoma cells. (A)The expression of PD-L1 remained no changes after PD-L2 knockdown in KHOS and U2OS cells, which was detected by western blot. (B) ROCK2 expression was inhibited after PD-L2 knockdown in KHOS and U2OS cells (C) Western blot analysis was used to evaluate the expression of LC3 and p62 after beclin1 knockdown which markedly decreased autophagy.

Figure S2: Effect of PD-L2 expression recovery on proliferation, migration, invasion and autophagy of osteosarcoma cells. (A) Cell proliferation, migration and invasion of KHOS cells after PD-L2 expression recovery were determined by cell colony formation and transwell assays. (B) The PD-L2 expression recovery increased MMP-9 and snail levels and induced EMT in shPDL2-KHOS cells. (C) The PD-L2 expression recovery increased p-LIMK2, p-cofilin, RGMb, neogenin and BMPR2 expressions in shPDL2-KHOS cells. (D) Western blot analysis was used to evaluate the expression of LC3, beclin1 and p62 after PD-L2 expression recovery. (E) Expression recovery of PD-L2 markedly increased autophagy. Data are presented as the mean ± S.D. **P<0.01, ***P<0.001.

Figure S3: The quantification of western blot results in Figure 4. (A) The quantification of western blot results in Figure 4A. (B) The quantification of western blot results in Figure 4B. (C) The quantification of western blot results in Figure 4D. (D) The quantification of western blot results in Figure 4E. (E) The quantification of western blot results in Figure 4G. Data are presented as the mean ± S.D. **P<0.01, ***P<0.001.

Figure S4: The quantification of western blot results in Figure 5, 6 and 7. (A) The quantification of western blot results in Figure 5C. (B) The quantification of western blot results in Figure 6A. (C) The quantification of western blot results in Figure 6B. (D) The quantification of western blot results in Figure 6C. (E) The quantification of western blot results in Figure 7F. Data are presented as the mean ± S.D. **P<0.01, ***P<0.001.

Figure S5: The quantification of western blot results in Figure S1 and S2. (A) The quantification of western blot results in Figure S1. (B) The quantification of western blot results in Figure S2B. (C) The quantification of western blot results in Figure S2C. (D) The quantification of western blot results in Figure S2D. Data are presented as the mean ± S.D. **P<0.01, ***P<0.001.
